# Fractal Analysis of Four Xerogels Based on TEGylated Phenothiazine and Chitosan

**DOI:** 10.3390/gels9060435

**Published:** 2023-05-25

**Authors:** Maria-Alexandra Paun, Mihai-Virgil Nichita, Vladimir-Alexandru Paun, Viorel-Puiu Paun

**Affiliations:** 1 School of Engineering, Swiss Federal Institute of Technology (EPFL), 1015 Lausanne, Switzerland; maria_paun2003@yahoo.com; 2 Division Radio Monitoring and Equipment, Section Market Access and Conformity, Federal Office of Communications (OFCOM), 2501 Bienne, Switzerland; 3 Doctoral School, Faculty of Applied Sciences, University Politehnica of Bucharest, 060042 Bucharest, Romania; mihai_nichita9@yahoo.com; 4 Five Rescue Research Laboratory, 75004 Paris, France; vladimir.alexandru.paun@ieee.org; 5 Physics Department, Faculty of Applied Sciences, University Politehnica of Bucharest, 060042 Bucharest, Romania; 6 Academy of Romanian Scientists, 50085 Bucharest, Romania

**Keywords:** TEGylated phenothiazine, chitosan, SEM images, fractal analysis, fractal parameters

## Abstract

The present article describes novel massive materials (in the solid phase) based on TEGylated phenothiazine and chitosan that possess great capability to recover mercury ions from constituent aqueous solutions. These were produced by chitosan hydrogelation accompanied by formyl subsidiary item of TEGylated phenothiazine, attended by lyophilization. The delineation and structure description of the obtained material or supramolecular assembly were realized by FTIR (Fourier transform infrared) spectroscopy, X-ray diffraction, and POM (Polarized Light Optical Microscopy). The morphology of their texture was kept under observation by SEM (Scanning Electron Microscopy). The obtained SEM images were evaluated by fractal analysis. The fractal parameters of interest were calculated, including the fractal dimension and lacunarity.

## 1. Introduction

The pollution of the ambient environment by heavy metals is a crucial concern worldwide, which seriously impacts animals’ and human’s general state of health [1]. Heavy metals are considered to be perilous pollutants because they are not biodegradable and pollute the air, water, and soil by contamination. These ones have a high penetration rate in the trophic stereotype (food chain) and, over time, in the human body. While several metals, such as chromium, copper, manganese, molybdenum, selenium, and zinc are simply necessary in daily diet, however in reduced quantity, an overexposure determines major intoxication followed by organ deterioration in the long-term period, particularly for children and adolescents [2]. Average concentrations (μg/gHb) in the erythrocytes (or red blood cells) are equal to 0.32 ± 0.16 (for Cr), 38.8 ± 6.68 (for Cu), 1.45 ± 0.36 (for Mn), 0.3 ± 0.04 (for Mo), 8.63 ± 2.30 (for Se), and 0.65 ± 0.10 (for Zn), in tested human subjects with low physical training levels. Other metals, for example cadmium, lead, mercury, and arsenic, were identified as eminently perilous for human body and health, even in low quantities. The geometric averages of cadmium, lead, mercury and inorganic arsenic in the blood are equal to 0.09 µg/L (max 0.26 µg/L) for Cd, 9.9 µg/L (max 42 µg/L) for Pb, 0.70 (max 2.4 µg/L) for Hg and 6.1 µg/L (max 10 µg/L) for iAs. The Centre for Disease Control (CDC), U.S. Food and Drug Administration, Joint Food, World Health Organization (WHO), Agricultural Organization (FAO) and the US Environmental Protection Agency (EPA) have decided their inclusion on the carcinogenic active agents list by all health agencies [3,4].

As regards, heavy metal contamination is closely related to global industrial development, which cannot be suspended. As a consequence, the world’s interstate agencies included these metallic compounds on the problematic chemical substances list, which require propriety monitoring and confirming the recommended maximal levels, both in water and soils [5,6]. Among these toxic/poisonous metals, mercury is particularly most perilous because it sublimates very easily, contaminates the air, is effortlessly stored in potable waters and soil, and is a tenacious contamination source [7]. In living organisms, mercury denatures the proteins and kills living cells, especially nervous system cells, the neurons [8]. For this reason, advisable mercury concentration values are very small; more precisely, they are limited below values of 2 ppb [9]. In the circumstances described above, the attention of specialists was concentrated on the discovery and improvement of materials that have the ability to identify and recuperate mercury from the ambient environment and corporeality (human body) [10,11,12,13,14,15,16].

Chemical substances generically named as xerogels are a gel type naturally found in the solid phase, which currently have properties such as a superior porosity and significant surface in coincidence with remissivity of the pore dimensions [17,18]. The present study is based on the new solid materials found on chitosan and TEGylated phenothiazine, which present a large capacity to recuperate mercury ions located in aqueous (water) solutions. Among these chemical substances presented above, we mention with primacy chitosan. Chitosan is a special biopolymer considering that it is abundant in nature, is positively electrically charged (cationic), has very low degree of toxicity, is immunodeficient (non-immunocompetent), and is essentially sustainable in an unlimited manner [19,20].

Fractal analysis is a quantitative method of image evaluation that is based on three established parameters, which are considered to be fractal dimension, lacunarity and succolarity [21]. The effective structure determining the properties of gel morphology is characterized by fractal dimensions deduced from the used theoretical model, which also suggests that the size of primary flocs building fractal structures is one of the important factors that determine the linear viscoelastic properties of the gels. The fractal dimension is the measure that discriminates how much a geometric object fills the space that includes it. Fractal dimension is an established quantity that does not amend with the scale, neither with applied translation or rotation procedure [22]. Lacunarity is the one which determines the measurements of the holes’ dimension and frequency on the picture. Succolarity measures in what quantity a well-determined fluid can flow over a picture, considering the set of pixels as a barrier with a definitive exact color (black or white, for example) on 2D picture evaluation.

## 2. Theoretical Part

In fractal theory, the fractal dimension, lacunarity, and succolarity notions are usually utilized to characterize and evaluate the structural information of the pore/hole system in the material. Still, the practical application and calculation of the three fractal parameters is difficult because of the complex definitions and laborious methods of computation. In this subchapter, we mainly introduce the classic definitions and physical meanings of these fractal structural parameters and the calculation method based on the box-counting procedure from pictures. In addition, several examples of the application of fractal parameters in physical property modeling, natural fracture characterization, and permeability prediction of the analyzed compounds are presented. These results can illustrate well the functions of the fractal dimension, lacunarity, and succolarity for the description of complexity and heterogeneity degree, as well as the anisotropy of the material’s body structure and porous media.

### 2.1. Fractal Dimension

The fractal dimension is the essential fractal parameter to depict a complex system, from the fractal point of view. It is a measure to reflect the space availableness (3D), the coverage with differentiable curves of the contour of some surfaces (2D) or the complex bodies’ nonuniformity. Referring to porous media, the fractal dimension is utilized in a quantitative manner, to characterize the statistical repartition of the orifices/holes dimension, the porous surfaces (with pores on them) rugosity, and the streamline outline curvature [22]. We still have to say here that, while distinct definitions generate dissimilar fractal dimension values, the fractal dimension is the ordinary procedure to exactly report the distribution of the pore/particle dimension of the porous media pictures. We will now discuss the basic relationship of the fractal scaling convention among the pore/particle collected number, noted *M*(*ɛ*), and the pore/particle caliber noted *ɛ*.

This can be written as the following relation:

(1) $$M\left(\varepsilon \right)\propto \varepsilon^{D_{f}}$$

wherein *D_f_* is a natural fractal dimension of the considered porous space. The fractal dimension can be considered, from a mathematical point of view, also as a measure of how all details in the fractal change with scale. There is also the problem of knowing, when the fractal object is projected against a grille, how many elements the fractal includes/covers as its elements’ numbers grow. We mention that the fractal dimension cannot have an integer value, as it is a fractional number, to be precise.

It is important to remember three important statistics ascertained to be the correct measure of the fractal dimension [23]. These are the information dimension, the correlation dimension, and the box-counting dimension, the last being the most frequently used calculation technique. To calculate the fractal dimension value by the box-counting method, we split the fractal (3D) space into hypercubes with the side lengthiness equal to *r*. Considering that *N*(*r*) is the hypercubes number engaged by the fractal geometric points, the box counting fractal dimension is computed to be [24]:

(2) $$D_{bc}=\underset{r\to 0}{\lim}\frac{logN\left(r\right)}{logr}$$

### 2.2. Lacunarity

The word “lacunarity” refers in a literary way to a lacuna or hollow, as acquired from the word “lake”. However, in lexical consideration, it has been differently defined as being denoted by words such as inhomogeneity, gappiness or translational (2D) and rotational (3D) invariance. Currently denoted as *Λ* in FracLac software, the lacunarity relates to both holes’ evidence and heterogeneity measure, equally [25,26].

Lacunarity and fractal dimension are in close communion with each other, thus making possible a good understanding of the fractal object surface morphology with its pores (holes, orifices), entirely. In particular, it refers to the balance between the homogeneity and inhomogeneity of the texture in an integrative version, with an emphasis on the holes’ (pores) statistics and their caliber as repartition function, of all things. In fractal analysis theory, the lacunarity notion construes/renders mathematically the measure of current holes (named porous texture) or “true texture” radiography [27]. We find that the observed inhomogeneity degree as well as rotational (3D) and translational (2D) invariance of the picture surface (where reduced lacunarity assumes the proof of image homogeneity), also confirm that rotating the image amends the given context in a non-significant way.

The mathematical equations that govern this process are presented below.

(3) $$\Lambda \left(\varepsilon \right)=\frac{Z^{\left(2\right)}}{{\left(Z^{\left(1\right)}\right)}^{2}}$$

(4) $$Z^{\left(1\right)}=\underset{\varepsilon }{\sum }P•Q\left(P,\varepsilon \right)$$

(5) $$Z^{\left(2\right)}=\underset{\varepsilon }{\sum }P^{2}•Q\left(P,\varepsilon \right)$$

(6) $$Q\left(P,\varepsilon \right)=\frac{n\left(P,\varepsilon \right)}{{\left(M-\varepsilon +1\right)}^{2}}$$

In the equations highlighted above, the letters specified below signify the quantities described in continuation. The map dimension is *M*, the box dimension is *ε* and the box mass is equal to *P*. The *n*(*P*, *ε*) is the box number containing *P* pixels and probability *Q*(*P*, *ε*) is calculated via Equation (6). At the same time *P* • *Q*(*P*, *ε*) is the first moment and *P^2^* • *Q*(*P, ε*) is the second moment, while *Z*^(1)^ and *Z*^(2)^ are the sum of the first and second moments, computed by Equations (4) and (5), respectively. Equation (3) is the lacunarity value *Λ*(*ε*) of the box dimension *ε* dataset [28,29]. In comparison with the other fractal parameters, lacunarity is a counterpart to the fractal dimension, but in conjunction, they offer a complete description of fractal object texture. Its value is directly proportional to the quantity repartition of the gaps/orifices present in the material. In other words, if a fractal has large lacunas or holes, the lacunarity is particularly great. However, one can say that if a fractal is almost 2D translationally invariant, it has reduced lacunarity [30,31].

In conclusion, we can say that lacunarity measures the size and frequency of gaps/holes from a representative image.

### 2.3. Succolarity

Succolarity estimates the image percolation degree and how much a certain fluid may circulate/run through this picture, taking into consideration the pixels’ suite with a defined color (e.g., white or black pixels) as possible obstacles in the surface analysis of 2D images. The principal idea is that succolarity utilization is a necessary characteristic in the pattern recognition affair, especially in order to perceive genuine textures. To evaluate this, let us consider an image that respects the representativeness criteria. Assume that every pixel in its plan position may be regarded/thought about as empty (lack of mass for black pixels) or having an impenetrable mass for white pixels.

To calculate the succolarity value, we use the formula:

(7) $$\sigma \left(BS\left(k\right),dir\right)=\frac{\sum_{k=1}^{n}OP\left(BS\left(k\right)\right)\times PR\left(BS\left(k\right),pc\right)}{\sum_{k=1}^{n}OP\left(BS\left(k\right)\right)xm\times PR\left(BS\left(k\right),pc\right)}$$

where (*BS*(*k*)) is box size, *k* is the number of possible divisions of an image in boxes and *dir* is a direction, one of the known ones, right and left. *PR*(*BS*(*k*), *pc*) signifies the pressure above the box *k* centroid, on the considered scale. This can be achieved using the centroid coordinates, more precisely on *x* (in the horizontal case) or else *y* (in vertical case) [32]. Let us do a simulation now of the evacuating or percolation capability of a fluid through the picture. The initial image was explored inundated in vertical plan (from bottom to top and from top to bottom) and in a horizontal plan (from left to right and from right to left). In addition, other directions may be utilized to generate various succolarity values of images, if are representative, naturally.

The importance of succolarity, different from fractal dimension and lacunarity, necessary to highlight different fractal properties, is thus demonstrated. The succolarity [33] denotes a particular flow ability that allows crossing the set. Technically speaking, a succolarity reported on fractal dynamic sets is defined as the number evaluation of filaments that allows the percolation phenomenon or, in the same measure, to flow through. The latter is not suitable for the evaluation of SEM images and therefore will not be used in continuation as a fractal parameter of interest.

Note, the three independent fractal parameters (fractal dimension, lacunarity and succolarity) are important characteristics that examine different picture aspects in a subtle complementary manner. Thus, there can be two images that can set forth the identical fractal dimension, but distinct lacunarity, or identical lacunarity, but distinct succolarity, and even a combination of the outcomes is possible.

## 3. Results and Discussion

### 3.1. Morphology Notions

**Figure 1:**
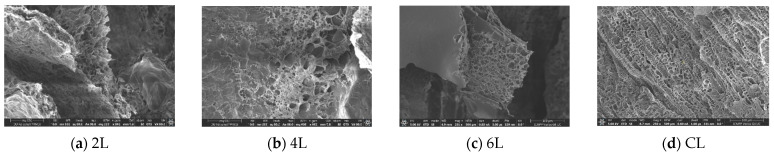
Four typical SEM images of the xerogels: (**a**) SEM-2L, (**b**) SEM-4L, (**c**) SEM-6L, (**d**) SEM-CL.

Known under this name in specialized literature, the xerogels manifested a similar texture of sponge-type morphology [34], with interconnected structural orifices (holes) and a polymorphous pores repartition with the included diameter in the interval 2 μm to 35 μm (Figure 1). Whilst the other authors report on the same subject the increase of the hole’s diameter as the reticular degree has diminished, no such a tendency was noticed for these specimens. This is most likely due to the fact that the imination degree in the hydrogel situation/condition was not sufficiently large to command the morphology, and thus the water congelation anterior to the lyophilization procedure gamed a decisive role. More, the displacement of the imination equilibrium to the chemical compounds at the time of lyophilization simply consolidated the morphology modeled in the congelation stage. In addition, the morphology was affected by the particular sublimation quota of water/acetone crystallites in the frosty hydrogels. The reduced density and freezing time of acetone, generated its rapid sublimation in comparison to water, dictating congestion of the hydrophobic phenothiazine items on the superficial appearance of xerogel outer veneers, composing a so-called thin film/layer.

### 3.2. Fractal Analysis of Scanning Electron Microscope Pictures

We will show the connection between the fractal analysis and the performance of the material in two examples. The first refers to the scaling behavior of gel elasticity. In theory, the gel network is considered a closely packed fractal flocs with the fractal dimension of *d*. The elastic properties of a floc are dominated by its effective backbone, which can be approximated as a linear chain of springs. The elastic constant (*K*) of the individual flocs is inversely related to their size (*l*). Since fractal flocs are considered scale invariant, the size of the flocs *l* is related to the volume fraction (*φ*) as $l\propto \varphi^{1/\left(d-3\right)}$. The second example refers to cluster–cluster aggregation. The process of colloidal aggregate formation has also been successfully investigated based on fractal considerations. Fractal growth models have been applied to the aggregation process of particles. In the cluster–cluster aggregation process, diffusing particles in a certain medium stick to one another at contact in a random way with probability *p*.

**Figure 2:**
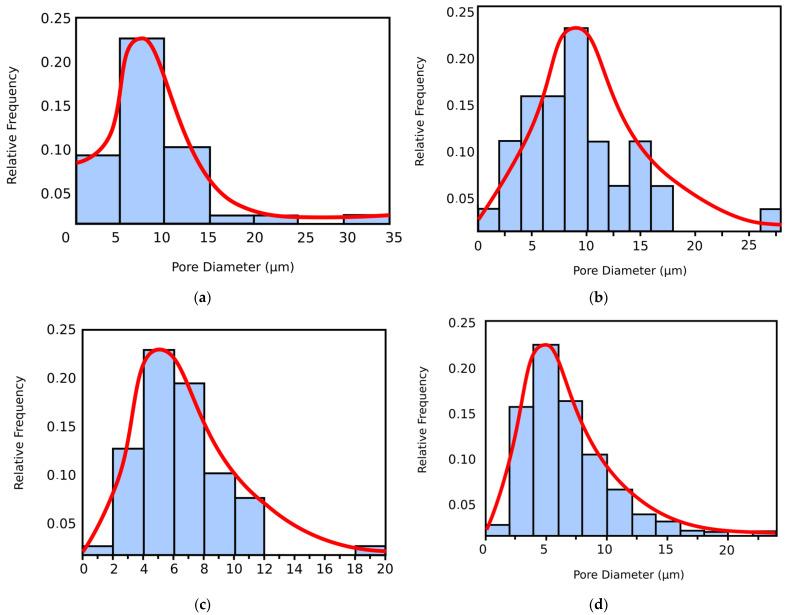
The histograms of pore dimension from SEM images, (**a**) SEM-2L, (**b**) SEM-4L, (**c**) SEM-6L, (**d**) SEM-CL.

The achieved hydrogels were proven to be transparent materials, and soft materials, which performed with maximum success in the test of inverted tube, and through the lyophilization process, porous solid materials were produced. The hydrogels acquired were further used, and the corresponding xerogels obtained by lyophilization have been noted with 2L, 4L, and 6L indicators. For a precise comparison with a consecrated xerogel, a chitosan xerogel reference was made ready in the identical conditions as the hydrogels aforesaid and noted with the CL indicator. In Figure 1, representative SEM typical pictures are visible [7]. More precisely, there are four SEM photographic images of four samples from those chemically obtained, representing four distinct xerogels, respectively noted with 2L, 4L, 6L and CL indicators. The SEM pictures scale bars, from the lower right side, in Figure 2 (a) SEM-2L; (b) SEM-4L; (c) SEM-6L and (d) SEM-CL, measure 100 microns for each [7].

Figure 2 shows the histograms of pore dimension (on the abscissa) from SEM images (Figure 1). For the four SEM images, the surface pores diameter is from 2 μm to almost 35 μm. On these PSD histograms are overwritten the PSD curves in red color.

#### 3.2.1. Fractal Parameters of 2L Image

**Figure 3:**
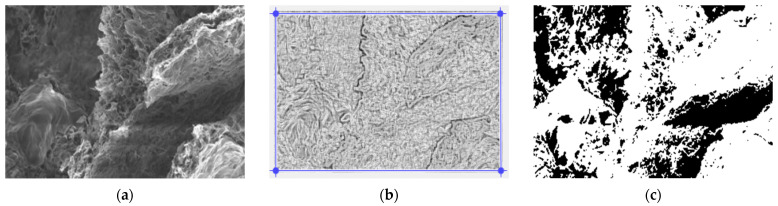
(**a**) Original 2L image, (**b**) Mask of 2L image, (**c**) The binarized version of the 2L image.

In Figure 3, we have the two phases of 2L original image processing and the fractal analysis techniques, such as the mask image version and binarized version, respectively, for the calculation of the fractal parameters. The threshold, above which the binarization of the 2L image was performed, is 77.

**Figure 4:**
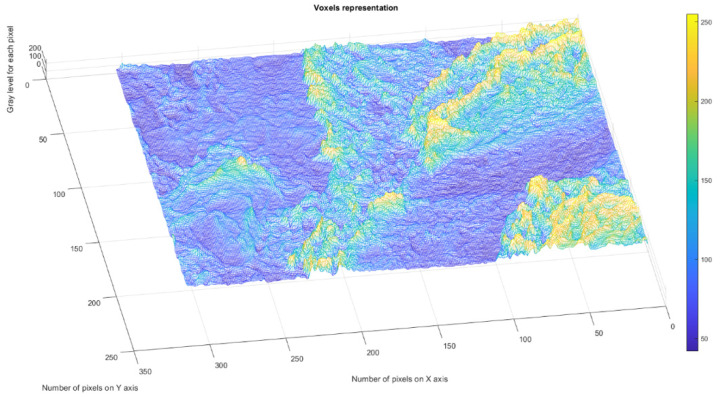
3D Voxels graphical representation of 2L image.

Figure 4 presents the voxels of the evaluated 2L picture, more precisely a 3D graphical portrayal/depiction, with the gray level on the oz axis, while the suitable number of pixels together with their position are marked on the other two plane axes, ox and oy, respectively [35].

**Figure 5:**
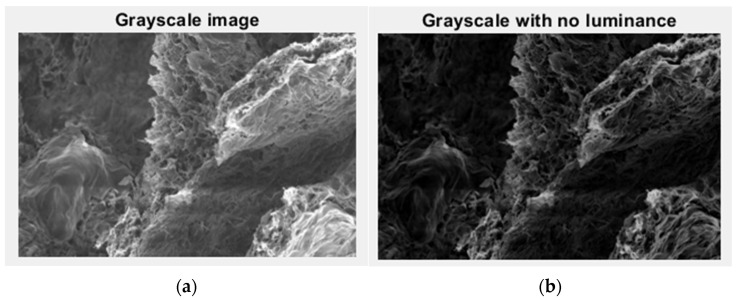
Grayscale versions of the 2L image: (**a**) with luminance, (**b**) without luminance.

In Figure 5, we have the two phases of 2L original image processing and the fractal analysis techniques, such as the gray scale with luminance version and the gray scale without luminance version, respectively, utilized for the calculation of the fractal parameters.

**Figure 6:**
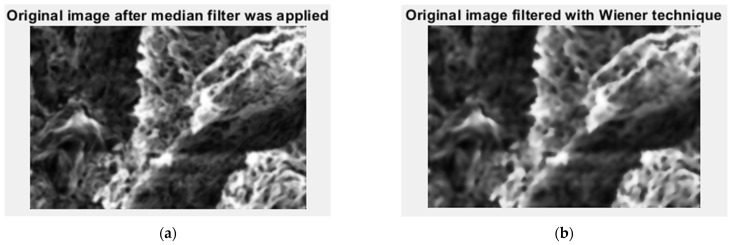
Filtered version of the 2L image after (**a**) median filter, (**b**) Wiener technique.

In Figure 6, we have the two phases of 2L original image processing as the filtered image version and the Wiener technique version, respectively, utilized for the calculation of the fractal parameters.

**Figure 7:**
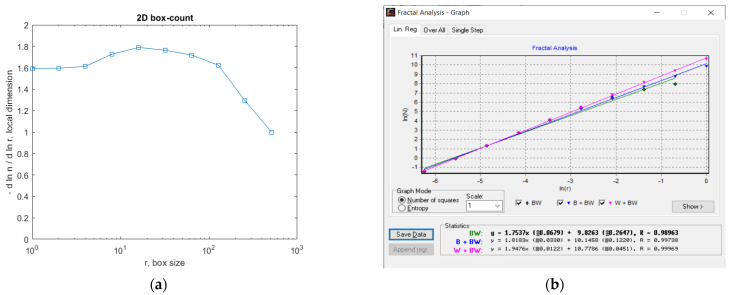
(**a**) Fractal local dimension for 2L image, (**b**) HarFA program for the 2L image.

In Figure 7, the fractal local dimension by box-counting algorithm in (a) and verification of the results with the HarFA (Harmonic and Fractal Image) program in (b), for the 2L image are presented [36].

In Figure 7a, the (2D) graphic to establish the fractal local dimension for the 2L image, the function of the box size *r*, by the boxes-counting procedure, is presented.

As a numerical appreciation upshot of the selected 2L picture, performed via the fractal analysis software developed by the authors, the values of Fractal Dimension FD1 = 1.604 and FD2 = 1.596, Standard Deviations *s*_1_ = ±√(σ^^2^) = ±0.2798 and *s*_2_ = ±√(σ^^2^) = ±0.0460, as well as Lacunarity value *Λ* = 0.0402, were estimated, seen in Table 1.

In the table above, the following notations were utilized:

- FD1-Fractal dimension with quadratic mask
- Standard deviation 1-Standard deviation with quadratic mask
- FD2-Fractal dimension with a rectangular mask
- Standard deviation 2-Standard deviation with a rectangular mask

Table 1 is a table with all the values of the fractal parameters obtained from the processing of the 2L image.

#### 3.2.2. Fractal Parameters of 4L Image

**Figure 8:**
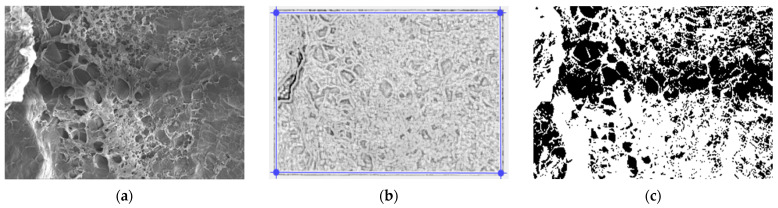
(**a**) Original 4L image, (**b**) Mask of 4L image, (**c**) The binarized version of the 4L image.

In Figure 8, we have the two phases of 4L original image processing and the fractal analysis techniques, such as the mask image version and binarized version, respectively, for the calculation of the fractal parameters. The threshold, above which the binarization of the 4L image was performed, is 100.

**Figure 9:**
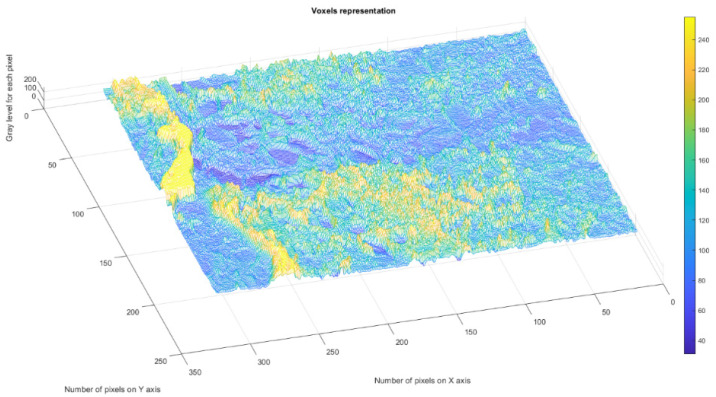
3D Voxels graphical representation of 4L image.

Figure 9 presents the voxels of the evaluated 4L picture, more precisely a 3D graphical portrayal/depiction, with the gray level on the oz axis, while the suitable number of pixels together with their position are marked on the other two plane axes, ox and oy, respectively [35].

**Figure 10:**
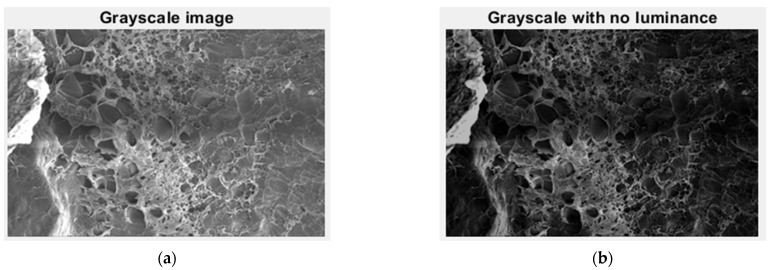
Grayscale versions of the 4L image: (**a**) with luminance, (**b**) without luminance.

In Figure 10, we have the two phases of 4L original image processing and the fractal analysis techniques, such as the gray scale with luminance version and the gray scale without luminance version, respectively, utilized for the calculation of the fractal parameters.

**Figure 11:**
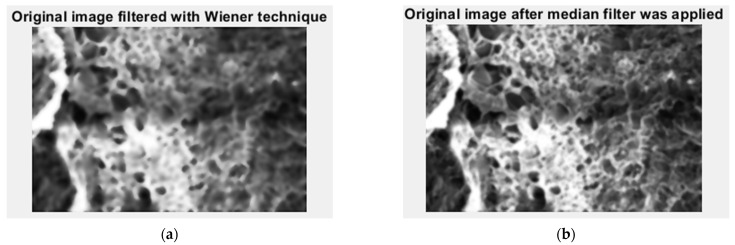
Filtered version of the 4L image after (**a**) median filter, (**b**) Wiener technique.

In Figure 11, we have the two phases of 4L original image processing as the filtered image version and the Wiener technique version, respectively, utilized for the calculation of the fractal parameters.

**Figure 12:**
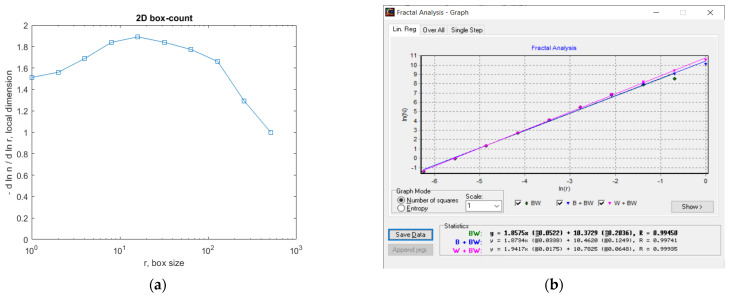
(**a**) Fractal local dimension for the 4L image, (**b**) HarFA program for the 4L image.

In Figure 12, the fractal local dimension by box-counting algorithm in (a) and verification of the results with the HarFA program in (b), for the 4L image are presented [36].

In Figure 12a, the (2D) graphic to establish the fractal local dimension for the 4L image, the function of the box size *r*, by the boxes-counting procedure, is presented.

As a numerical appreciation upshot of the selected 4L picture, performed via the fractal analysis software developed by the authors, the values of Fractal Dimension FD1 = 1.668 and FD2 = 1.615, Standard Deviations *s*_1_ = ±√(σ^^2^) = ±0.3127 and *s*_2_ = ±√(σ^^2^) = ±0.1445, as well as Lacunarity value *Λ* = 0.0526, were estimated, seen in Table 2.

Table 2 is a table with all the values of the fractal parameters obtained from the processing of the 4L image.

#### 3.2.3. Fractal Parameters of 6L Image

**Figure 13:**
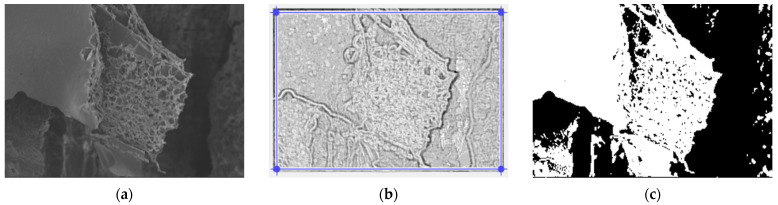
(**a**) Original 6L image, (**b**) Mask of 6L image, (**c**) The binarized version of the 6L image.

In Figure 13, we have the two phases of 6L original image processing and the fractal analysis techniques, such as the mask image version and binarized version, respectively, for the calculation of the fractal parameters. The threshold, above which the binarization of the 6L image was performed, is 75.

**Figure 14:**
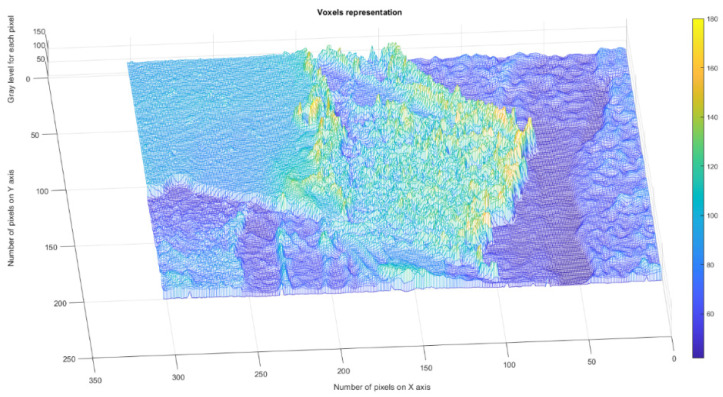
3D Voxels graphical representation of 6L image.

Figure 14 presents the voxels of the evaluated 6L picture, more precisely a 3D graphical portrayal/depiction, with the gray level on the oz axis, while the suitable number of pixels together with their position are marked on the other two plane axes, ox and oy, respectively [35].

**Figure 15:**
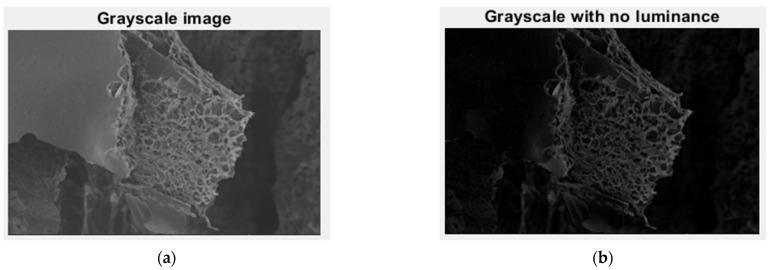
Grayscale versions of the 6L image: (**a**) with luminance, (**b**) without luminance.

In Figure 15, we have the two phases of 6L original image processing and the fractal analysis techniques, such as the gray scale with luminance version and the gray scale without luminance version, respectively, utilized for the calculation of the fractal parameters.

**Figure 16:**
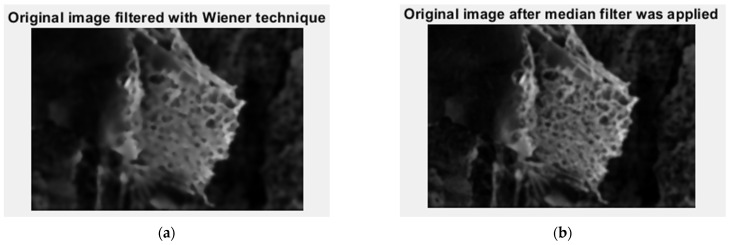
Filtered version of the 6L image after (**a**) median filter, (**b**) Wiener technique.

In Figure 16, we have the two phases of 6L original image processing as the filtered image version and the Wiener technique version, respectively, utilized for the calculation of the fractal parameters.

**Figure 17:**
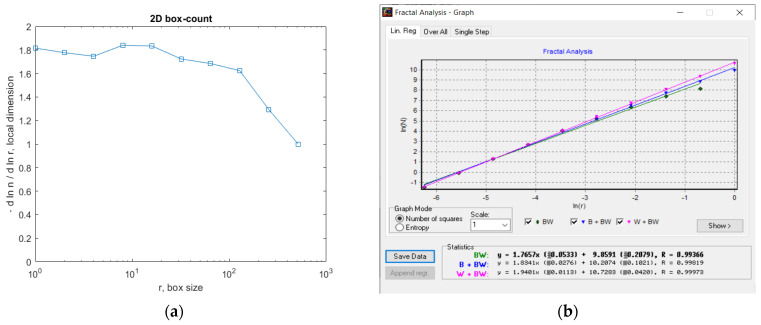
(**a**) Fractal local dimension for the 6L image, (**b**) HarFA program for the 4L image.

In Figure 17, the fractal local dimension by box-counting algorithm in (a) and verification of the results with the HarFA program in (b), for the 6L image are presented [36].

In Figure 17a, the (2D) graphic to establish the fractal local dimension for the 6L image, the function of the box size *r*, by the boxes-counting procedure, is presented.

As a numerical appreciation upshot of the selected 6L picture, performed via the fractal analysis software developed by the authors, the values of Fractal Dimension FD1 = 1.624 and FD2 = 1.615, Standard Deviations *s*_1_ = ±√(σ^^2^) = ±0.2947 and *s*_2_ = ±√(σ^^2^) = ±0.0298, as well as Lacunarity value *Λ* = 0.0381, were estimated as in Table 3.

Table 3 is a table with all the values of the fractal parameters obtained from the processing of the 6L image.

#### 3.2.4. Fractal Parameters of CL Image

**Figure 18:**
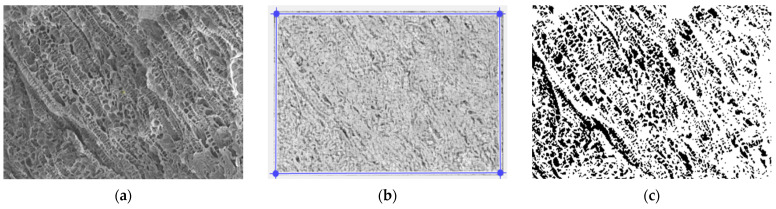
(**a**) Original CL image, (**b**) Mask of CL image, (**c**) The binarized version of the CL image.

In Figure 18, we have the two phases of CL original image processing and the fractal analysis techniques, such as the mask image version and binarized version, respectively, for the calculation of the fractal parameters. The threshold, above which the binarization of the CL image was performed, is 100.

**Figure 19:**
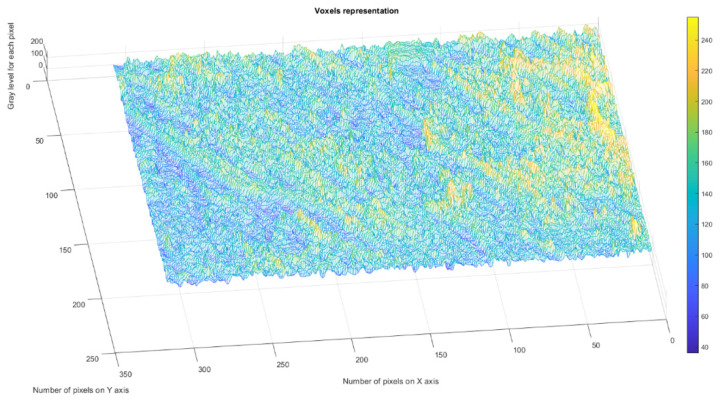
3D Voxels graphical representation of CL image.

Figure 19 presents the voxels of the evaluated CL picture, more precisely a 3D graphical portrayal/depiction, with the gray level on the oz axis, while the suitable number of pixels together with their position are marked on the other two plane axes, ox and oy, respectively [35].

**Figure 20:**
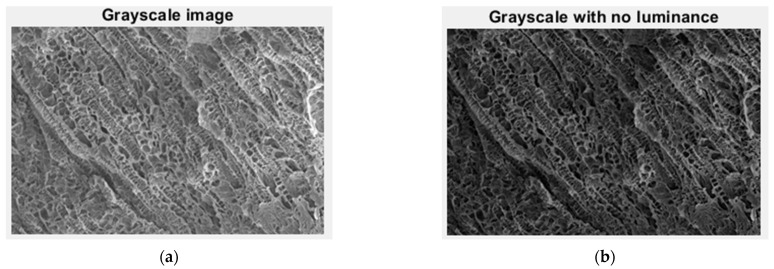
Grayscale versions of the CL image: (**a**) with luminance, (**b**) without luminance.

In Figure 20, we have the two phases of CL original image processing and the fractal analysis techniques, such as the gray scale with luminance version and the gray scale without luminance version, respectively, utilized for the calculation of the fractal parameters.

**Figure 21:**
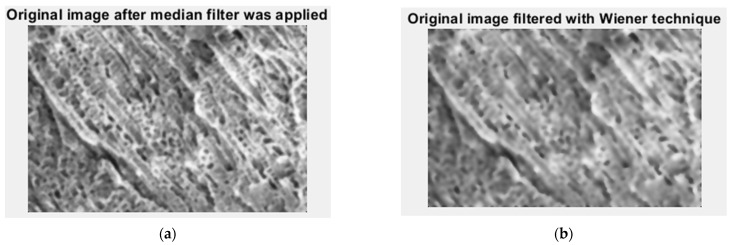
Filtered version of the CL image after (**a**) median filter, (**b**) Wiener technique.

**Figure 22:**
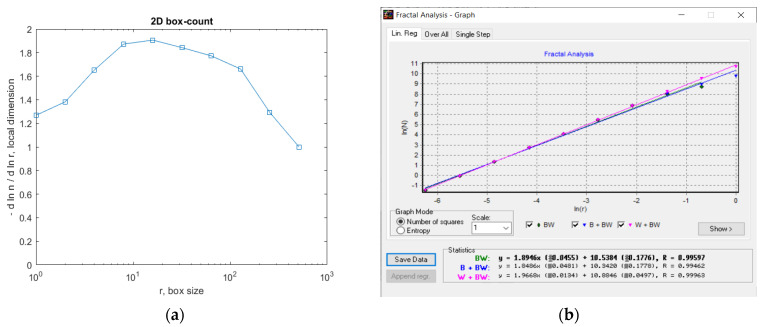
(**a**) Fractal local dimension for 6L image, (**b**) HarFA program for the CL image.

In Figure 21, we have the two phases of CL original image processing as the filtered image version and the Wiener technique version, respectively, utilized for the calculation of the fractal parameters. In Figure 22. the fractal local dimension by box-counting algorithm in (a) and verification of the results with the HarFA program in (b), for the CL image are presented [36].

In Figure 22a, the (2D) graphic to establish the fractal local dimension for the 2L image, function of the box size *r*, by the boxes-counting procedure, is presented.

As a numerical appreciation upshot of the selected CL picture, performed via the fractal analysis software developed by the authors, the values of Fractal Dimension FD1 = 1.678 and FD2 = 1.518, Standard Deviations *s*_1_ = ±√(σ^^2^) = ±0.3192 and *s*_2_ = ±√(σ^^2^) = ±0.3339, as well as Lacunarity value *Λ* = 0.0274, were estimated, seen in Table 4.

Table 4 is a table with all the values of the fractal parameters obtained from the processing of the CL image.

#### 3.2.5. Processing of Experimental Results. Discussions

The effective structure determining the properties of gels morphology is characterized by fractal dimensions deduced from the used theoretical model, which also suggests that the size of primary flocs building fractal structures is one of the important factors that determine the linear viscoelastic properties of the gels. Thus, for example, the values of fractal dimension d∼1.6–1.75 represent a material’s superior porosity, and the fractal dimension d∼1.8 is agreed in the case of diffusion-limited cluster-cluster aggregation.

The experimental data obtained, which are the subject of Table 5, were processed with appropriate calculation programs and then were represented graphically [31,37].

Table 5 is a table with all the values of the fractal parameters obtained from the processing of every analyzed image.

**Figure 23:**
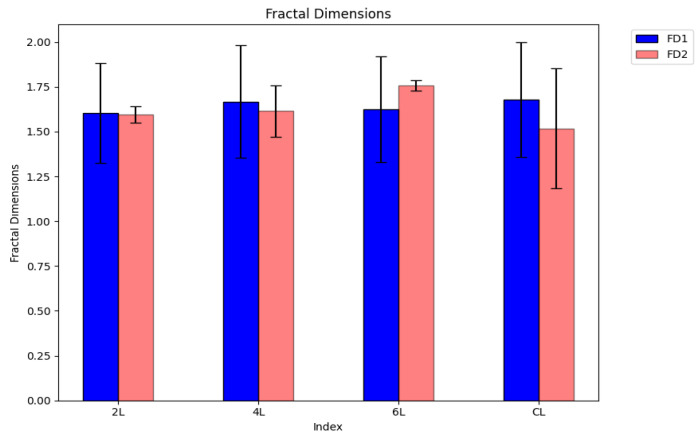
Fractal dimension histograms of the SEM images for four distinct chemical compounds.

The histograms of the fractal dimension of four distinct xerogels are presented in Figure 23. In this graph, the error bars of standard deviations for each individual SEM evaluated sample can also be seen.

The histograms colored in blue marked with FD1 represent Fractal dimension calculation with a quadratic mask, while the histograms colored in dark orange marked with FD2 represent fractal dimension calculation with a rectangular mask. It is observed that for the samples from the SEM images marked with 2L, 4L and CL, the fractal dimension for the calculation with a rectangular mask is smaller than the one calculated with the quadratic mask, while for the sample from the SEM image marked with 6L, the fractal dimension has inverted values for those two types of masks. The calculated values of the fractal dimension are in the range of 1.518 to 1.758, both calculated for a rectangular mask. The different values of the fractal dimension mean a lack of homogeneity of the pores in the four SEM-evaluated samples.

**Figure 24:**
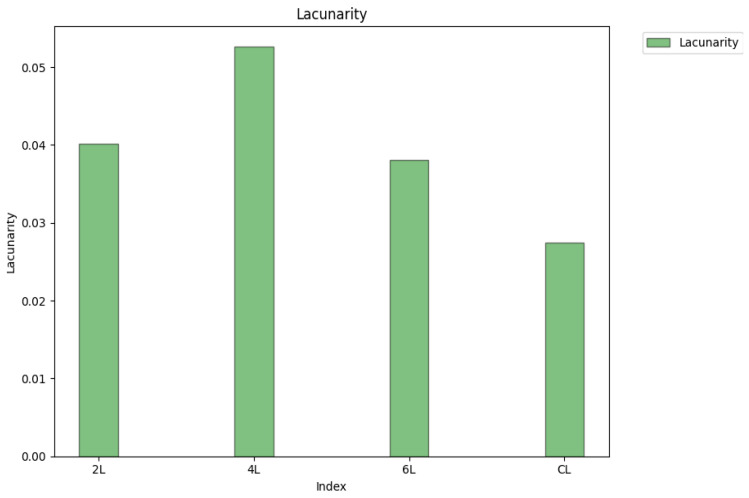
Histograms with the lacunarity value of the SEM images for four distinct chemical compounds.

The lacunarity histograms of four distinct xerogels are presented in Figure 24.

Note. Therefore, the SEM image of the sample marked CL, which has a lacunarity equal to 0.0274, has on average the smallest pores, while the SEM image of the sample marked 4L has on average the largest pores, at a lacunarity equal to 0.0526.

## 4. Conclusions

In the current paper, novel massive materials (in the solid phase under normal conditions of temperature and pressure) based on TEGylated phenothiazine and chitosan, some important chemical compounds that show a great capacity to recover the mercury ions from the constitutive aqueous solutions, are presented. The xerogels exhibited a sponge-like morphology type, which works with interconnected pores and a highly heterogeneous pore distribution with diameters ranging from 2 μm to 35 μm. Several important aspects of texture morphology are distinguished inside fractal analysis. Their texture morphology assessments, based on the fractal analysis of the SEM images, were performed accurately. The four SEM pictures indexed 2L, 4L, 6L, and CL of the different chemical formulations have been examined. The obtained results, respectively the values of the calculated fractal parameters, are the subject of Table 5, the two parameters of fractal geometry discussed here being fractal dimension and lacunarity. The fractal dimensions are in the range of 1.518 to 1.758, both values calculated for a rectangular mask. The fractal dimension values d∼1.6–1.75, such as those obtained by us, represent a material with a superior porosity, as expected. The lacunarity values are contained between 0.0274 and 0.0526. Intrinsically determined by the physical presence of pores in the tested samples, they are well surprised by the analyzed SEM images. According to the recognized theoretical assertions, patterns with bigger gaps (or pores) generally prove a higher lacunarity. Based on the values of the fractal parameters presented above, we can thus say that the xerogels obtained experimentally rise to the height of the expected qualities.

The work will be continued with the complex fractal analysis of the SEM images for the same xerogels. Furthermore, a multifractal model will be developed regarding the presence of mercury alongside the basic xerogel as its host, namely a theory of two bodies acting in solidarity (together, as one). All these things will be the subject of a future scientific paper.

## 5. Materials and Methods

### 5.1. Materials

The following materials such as reduced molecular weight chitosan, triethylene glycol monomethyl ether 97%, phenothiazine 98%, sodium hydride 95%, phosphorus (V) oxychloride 99%, and magnesium sulfate (MgSO4) 99.5%, have been acquired/bought from the Sigma-Aldrich Company(St. Louis, MO, USA). TEGylated phenothiazine refers to the fact that the phenothiazine heterocycle has been substituted with TEG (Triethylene Glycol), in other words, the phenothiazine core has a TEG chain attached. The chitosan molecular mass (198 kDa) was obtained by viscosity measurement founded on Mark–Houwink formula, with an Ubbelohde type viscometer. The acetylation degree (DA = 18%) was established from 1H-NMR. Acetone, dichloromethane (DCM) 99.5%, and dichloroethane (DCE) 99% were acquired from ROTH Company. Acetic acid and mercury (II) acetate were bought from VWR Company [7]. All resolvents and reagents were utilized as they were received.

### 5.2. Equipment and Methods

The spectra in the infrared domain were realized with the help of a Spectrometer of type FTIR Bruker Vertex 70 (Bruker Optics Company, 40 Manning Road, Manning Park, Billerica, MA, USA), operating in transmission regime, utilizing KBr granules, at normal temperature and pressure, by 2 cm^−1^ resolution. Origin8 software was utilized to process the recorded spectra. The NMR investigations were executed on the spectrometer of type Bruker Avance Neo (400 MHz) (International Equipment Trading Ltd., 955 Campus Drive, Mundelein, IL, USA) provided with a space probe-type instrument based on four 5 mm diameter cores and unbiased *z*-axis-gradient detection. The both spectra, photoluminescence, and UV-Vis absorption, were realized on a spectrophotometer of type PerkinElmer LS 55 (International Equipment Trading Ltd., 955 Campus Drive, Mundelein, IL, USA) and a spectrophotometer of type Agilent Cary 60 UV-Vis (Oxford Instruments Company, Abingdon, Oxfordshire, England) respectively, on solid specimens. The SEM pictures were produced with a Scanning Electron Microscope of type SEM EDAX—Quanta 200 (PHILIPS Company, Eindhoven, The Netherlands), at a smaller energy of 20 Kev for the electrons [7].

## Figures and Tables

**Table 1:** Fractal characteristics computation of picture 2L.

FD1	Standard Deviation 1	FD2	Standard Deviation 2	Lacunarity
1.604	±0.27987	1.596	±0.04607	0.0402

**Table 2:** Fractal characteristics computation of picture 4L.

FD1	Standard Deviation 1	FD2	Standard Deviation 2	Lacunarity
1.668	±0.3127	1.758	±0.1445	0.0526

**Table 3:** Fractal characteristics computation of picture 6L.

FD1	Standard Deviation 1	FD2	Standard Deviation 2	Lacunarity
1.624	±0.2947	1.758	±0.0298	0.0381

**Table 4:** Fractal characteristics computation of picture CL.

FD1	Standard Deviation 1	FD2	Standard Deviation 2	Lacunarity
1.678	±0.3192	1.518	±0.3339	0.0274

**Table 5:** Fractal characteristics computation of all images.

Index	FD1	Standard Deviation 1	FD2	Standard Deviation 2	Lacunarity
2L	1.604	±0.27987	1.596	± 0.04607	0.0402
4L	1.668	±0.3127	1.758	± 0.1445	0.0526
6L	1.624	±0.2947	1.758	± 0.0298	0.0381
CL	1.678	±0.3192	1.518	± 0.3339	0.0274

## Data Availability

The data used to support the findings of this study cannot be accessed due to commercial confidentiality.
